# The Role of Incubation Conditions in the Onset of Avian Myopathies

**DOI:** 10.3389/fphys.2020.545045

**Published:** 2020-09-18

**Authors:** Edgar Orlando Oviedo-Rondón, Sandra G. Velleman, Michael J. Wineland

**Affiliations:** ^1^Prestage Department of Poultry Science, North Carolina State University, Raleigh, NC, United States; ^2^Department of Animal Sciences, The Ohio State University, Columbus, OH, United States; ^3^Hatchery Consultant Inc., Raleigh, NC, United States

**Keywords:** incubation, myopathies, temperature, hypoxia, metabolism, chickens, ducks, turkeys

## Abstract

White striping, wooden breast, and spaghetti muscle have become common myopathies in broilers worldwide. Several research reports have indicated that the origin of these lesions is metabolic disorders. These failures in normal metabolism can start very early in life, and suboptimal incubation conditions may trigger some of the key alterations on muscle metabolism. Incubation conditions affect the development of muscle and can be associated with the onset of myopathies. A series of experiments conducted with broilers, turkeys, and ducks are discussed to overview primary information showing the main changes in breast muscle histomorphology, metabolism, and physiology caused by suboptimal incubation conditions. These modifications may be associated with current myopathies. Those effects of incubation on myopathy occurrence and severity have also been confirmed at slaughter age. The impact of egg storage, temperature profiles, oxygen concentrations, and time of hatch have been evaluated. The effects have been observed in diverse species, genetic lines, and both genders. Histological and muscle evaluations have detected that myopathies could be induced by extended hypoxia and high temperatures, and those effects depend on the genetic line. Thus, these modifications in muscle metabolic responses may make hatchlings more susceptible to develop myopathies during grow out due to thermal stress, high-density diets, and fast growth rates.

## Introduction

The growth, development, structure, and general metabolism of muscles in poultry species have been modified by genetic selection (Tesseraud et al., 2003; Petracci et al., 2014, 2019). These genetic changes probably resulted in modifications that affect the biochemical and sensory characteristics of meat. Havenstein et al. (1994a; 1994b; 2003a; 2003b) concluded that 85 to 90% of the differences between today’s broiler and the average chicken 60 years ago are due to genetic selection. In recent years, numerous studies showed that fast-growing genetic lines exhibited a high incidence of idiopathic myopathies and greater susceptibility to stress-induced myopathies that have significant implications on meat quality. Nowadays, the poultry industry observes an increased incidence of abnormal conditions such as deep pectoral myopathy also called green muscle disease (Kijowski et al., 2014), exudative meat or little water retention, wooden breast, striated breast and spaghetti muscle in broilers (Owens et al., 2009; Petracci and Cavani, 2012; Bailey et al., 2015; Russo et al., 2015; Petracci et al., 2019), white stripping in turkeys (Soglia et al., 2018) and myopathies in ducks (Gopalakrishnakone, 1986). Bailey et al. (2015) indicated the importance of understanding the environmental and management factors that contribute with more than 65% to the variance of incidence for striated breast and with more than 90% to the variation in the occurrence of wooden breast and deep pectoral myopathy in broilers.

An essential factor to consider in the management of poultry species is the incubation conditions during embryo development. Avian embryos exhibit phenotypic (De Smit et al., 2006; Branum et al., 2016) and metabolic (Loh et al., 2004; McNabb, 2006) developmental plasticity to temperature, humidity, oxygen, and CO_2_ concentrations among other environmental factors like light (Halevy et al., 2006b). Phenotypic plasticity includes all interactions of an organism’s genotype with its environment (Burggren et al., 2016).

Artificially incubated eggs are influenced by the temperature, ventilation, humidity, and turning that allow tissue growth, extraembryonic membrane development, gas exchange, and moisture loss (Decuypere and Michels, 1992; French, 1997; Molenaar et al., 2010; Boleli et al., 2016). However, the optimum parameters for all embryos in a machine are not the same due breeder genetics and age, egg composition and yolk size, eggshell and albumen properties, storage time and temperature of storage, among other factors (Molenaar et al., 2010; Yalçin et al., 2014; El Sabry et al., 2015; Nangsuay et al., 2017; Okur et al., 2018).

In large incubator machines, embryo development is primarily affected by the physical microenvironment around the egg (French, 1997; Boleli et al., 2016). The temperature sensed by the embryo depends on three factors: (1) the air temperature, (2) the heat exchange between the egg and its microenvironment depending on the speed of airflow, and (3) the heat production of the embryo that increases as it growths (Van Brecht et al., 2005). It has been demonstrated that any variation on incubation parameters especially the long-term high temperatures and low oxygen tension during the last incubation phases in the hatchers may affect negatively almost all tissues and organ development, including muscles (Hulet et al., 2007; Leksrisompong et al., 2007; Molenaar et al., 2010; Janisch et al., 2015; Boleli et al., 2016; Clark et al., 2017). The impact of those developmental changes has been observed during the life post-hatch in growth and meat quality parameters or the incidence and severity of myopathies. The objective of this publication is to present the scientific evidence that shows that incubation conditions may be an essential factor affecting the susceptibility of poultry to myopathies. The first part of this manuscript will describe the muscle development in avian embryos, its regulation, and the effects of the environment. The second portion will provide a brief description of myopathies with an emphasis on the wooden breast. The third section will discuss evidence that incubation can cause myopathy-like effects in chickens, turkeys, and ducks at hatch and processing age.

## Muscle Development in Avian Embryos

Understanding muscle development is essential to recognize the multiple effects of incubation conditions on the onset of myopathies. The development of skeletal muscle depends on myogenesis and, in part, also on adipogenesis from embryogenesis to adulthood. In the embryo, myogenesis starts with myogenic determination activating myoblasts that first proliferate and then differentiate and fuse into multinucleate fibers. Myofiber ontogenesis commences very early during embryonic life, with the presentation of two or three successive waves of myoblasts, which establish the origin of the diverse types of muscle fibers (Picard et al., 2002; Halevy, 2020).

### Muscle Cell Development

In the chick, embryonic myoblasts are most abundant on embryonic day 5 (ED5) during the primary wave of myogenic cells occurring between ED3 and ED7, whereas fetal myoblasts are most abundant between ED8 and ED12 in the second wave of myogenic cells that occurs between ED7 and ED16 (Stockdale, 1992; Picard et al., 2002). From ED15 onward, satellite cells, or also called adult myoblasts, can be distinguished by their morphology and location under the basal membrane of the myofibers (Hartley et al., 1992). These cells are the primary source of myogenic precursors in the post-hatch muscle (Schultz and McCormick, 1994); however, their numbers are reduced to less than 5% of total myofiber nuclei toward the end of the growth phase, and they become largely quiescent (Halevy et al., 2001, 2004). Nevertheless, satellite cells can reenter the cell cycle in response to several muscular stresses and go through proliferation, followed by the withdrawal from the cell cycle and fusion into existing or newly formed fibers (Schultz and McCormick, 1994; Hawke and Garry, 2001). Short-term changes in incubation temperature can stimulate the development of satellite cells during different time frames of embryonic development. This thermal stimulus has been successful when applied during the first 4 to 12 days of incubation (Maltby et al., 2004; Krischek et al., 2013a, b), in late-term embryos between ED16 and ED18 (Halevy et al., 2006a, c; Piestun et al., 2013) as well as by changes in environmental temperature during early post-hatch development (Halevy et al., 2001, 2006c). Those are the positive effects of incubation temperature on muscle development, but they only occur when applied in these embryonic developmental periods and during short time in what it has been called circadian incubation.

The muscle mass and intramuscular fat are both determined by cell numbers and unit cell size, mainly during embryo development in avian species (Picard et al., 2002). Cell numbers of adipocytes and muscle fibers increase during the embryonic period by hyperplasia, basically until hatch time (Smith, 1963; Sporer et al., 2011). Breast muscle organizational differences among breeds and between sexes begin to occur between ED20 and ED25 in turkeys (Velleman et al., 2007). While the average cross-sectional area and diameter of the fibers peaks at ED27 in ducks (Li et al., 2016). Thus, embryonic muscle development during the perinatal period has an essential impact on the post-hatch accumulation of muscle mass and its infiltration with intramuscular fat (Velleman, 2007). Studies of proteomics and metabolomics of the chicken breast muscle from ED12 to D14 (Liu et al., 2017) indicated that the growth of the embryonic muscle in Cobb chickens is mainly a result of sturdier hyperplasia process than hypertrophy. The extent of this hyperplasia is the major contributor to the post-hatchling excellent muscle mass accretion in fast-growing Cobb broiler chicks, which has been measured as a 35.9-fold change from D1 to D14. The main time for intramuscular fat accumulation is from ED17 to day 1 (D1) post-hatch in chickens (Liu et al., 2017), and in mule ducks, the highest level was D1 (Chartrin et al., 2007). Most of the hyperplasia and intramuscular fat accretion happens during the time of higher oxygen demand.

Myogenesis is governed by a family of proteins known as myogenic regulatory factors (MRFs). These basic helix loop helix (bHLH) transcription factors act sequentially in myogenic differentiation. The vertebrate MRF family members include MyoD1, Myf5, myogenin, and MRF4 (Myf6). These transcription factors work together with the myocyte enhancer factor-2 proteins. MyoD1 and myogenin are successively expressed only in activated satellite cells (Hernández-Hernández et al., 2017). The paired-box transcription factor Pax7 is selectively expressed in quiescent and proliferating satellite cells and is essential in their self-renewal. Pax7 is a marker of myogenesis during post-hatch muscle growth. Its expression is preserved by satellite cells in the adult chicken muscle (Halevy et al., 2004; Allouh et al., 2008).

Hypoxia during embryonic development is a potent inhibitor of myoblast differentiation decreasing expression of MyoD, Myf5, myogenin, and myosin heavy chains, which hampers the formation of multinucleated myotubes (Beaudry et al., 2016; Yang et al., 2017). The impact of hypoxia will depend on timing during the muscle maturation process (Halevy, 2020) and the severity of oxygen deprivation (Beaudry et al., 2016). Myoblast can recover their capacity to proliferate and differentiate when normal oxygen levels are restored (Beaudry et al., 2016). This process is mediated by the hypoxia-inducing factor (HIF) proteins with his three α isoforms HIF-1α, HIF-2α, and HIF-3α. The balance between HIF-1α and HIF-2α is important for muscle regeneration and satellite cell self-renewal (Yang et al., 2017). Embryos may overcome hypoxia challenges pre-hatch, but mechanisms for post-hatch muscle regeneration could be affected in muscles that have suffered injuries during extended hypoxia. In summary, all these regulatory factors can be affected by incubation conditions leading to different phenotypic and metabolic traits in muscles (Al-Musawi et al., 2012; Harding et al., 2015; Malila et al., 2019).

Satellite cell proliferation and differentiation are affected by several growth factors and hormones. One of them is the muscle-secreted insulin-like growth factor I (IGF-I) isoform that increases myofiber hypertrophy (Adams et al., 2000). Temperature and oxygen concentration affect levels and cellular signaling of IGF in muscles during the perinatal period (Duan et al., 2010). Halevy et al. (2001) demonstrated that elevated levels of IGF-I in the chick muscle in response to mild heat stress (37°C) for 24 h at D3 post-hatch, resulted in greater muscle cell proliferation, differentiation and continuous muscle growth in broilers. On the contrary, lower IGF-I muscle concentrations and more significant myostatin production caused by high levels of glucocorticoids have been both confirmed to contribute to the muscle atrophy of fast-twitch or type II muscle fibers, present in breast muscle. Glucocorticoid levels are increased from ED16 until the day of hatch (Wise and Frye, 1973; Tona et al., 2003b). Higher concentrations of glucocorticoids have been observed in embryos from stored eggs (Tona et al., 2003b), during hypoxia (Giussani et al., 2007; Tong et al., 2015) or on embryos exposed to higher temperatures (von Blumröder and Tönhardt, 2002). Hypoxia also alters the cellular response to IGF signaling by reprogramming the intracellular signal transduction network (Duan et al., 2010).

### Myogenesis Regulation and Environmental Effects

Research on thermal manipulation of chick embryos at critical phases of development has been conducted to improve long-term physiological responses, such as thermoregulation and muscle development (Yahav et al., 2004a, b; Piestun et al., 2008). These thermal manipulations frequently caused noteworthy decay in plasma thyroid hormone concentration (Yahav et al., 2004a, b; Piestun et al., 2008), which has been linked to myopathies and other metabolic disorders (Valentin, 2017). In contrast, thermal manipulations for 3 h at 38.5°C or 39.5°C have been sufficient to stimulate muscle development when applied during the period of maximum satellite cell expansion in chick embryos ED16 to ED18. The result in broilers at 42 days of age has been increased *Pectoralis* muscle weight compared to controls. However, changes in machine temperature during other embryonic periods had no effect. Despite the potential benefits of thermal conditioning, these short periods of thermal modifications are still not standard in the poultry industry worldwide, and constant heat stress due to multi-stage incubation or inadequate management of single-stage profiles are more common (Molenaar et al., 2010, 2013; Boleli et al., 2016).

Significant adverse effects on meat yield and muscle development have been observed with continuous or long-term (more than 6 h) cyclic high temperatures (>38°C) during incubation (Hulet et al., 2007; Naraballobh et al., 2016a, b; Clark et al., 2017). High incubation temperatures increase oxygen consumption of embryos during the late endothermic phase of incubation that occurs in the 3 to 4 days before hatch (Janke et al., 2002; Mortola and Labbè, 2005; Molenaar et al., 2010, 2013). Consequently, high incubation temperatures in the plateau stage (ED17 to ED21) of oxygen consumption (Rahn, 1981) makes embryos more susceptible to all adverse developmental effects of hypoxia during this last phase (Wineland et al., 2006a, b; Oviedo-Rondón et al., 2008a, b, 2009a,b).

Embryos from precocial poultry species face some hypoxia during the hatching process, but the extent of this phase may have detrimental effects on the development of almost all organs and tissues (Tona et al., 2003a; Mortola and Labbè, 2005; De Smit et al., 2006). During this late phase of incubation, the eggshell conductance, frequently nominated as ***G***, becomes very important for embryo respiration (Rahn, 1981; Mortola and Labbè, 2005; Oviedo-Rondón et al., 2008a, b, 2009a,b). The ***G*** is the eggshell capacity to passively transfer gasses from the environment to the embryo that can also be measured indirectly by loss of metabolic water and gasses from inside the egg to the external environment (Rahn, 1981). Eggs may vary on ***G*** depending on specie, genetics, breeder age, and egg size (Rahn, 1981; Visschedijk et al., 1985; Nangsuay et al., 2017). Eggs with reduced ***G*** may face more severe hypoxia. Taking into consideration that recent findings have linked myopathy onset with hypoxia (Malila et al., 2019), the factors that cause hypoxia during life should receive attention to determine strategies to minimize myopathy incidence.

## Breast Myopathies in Avian Species

**FIGURE 1:**
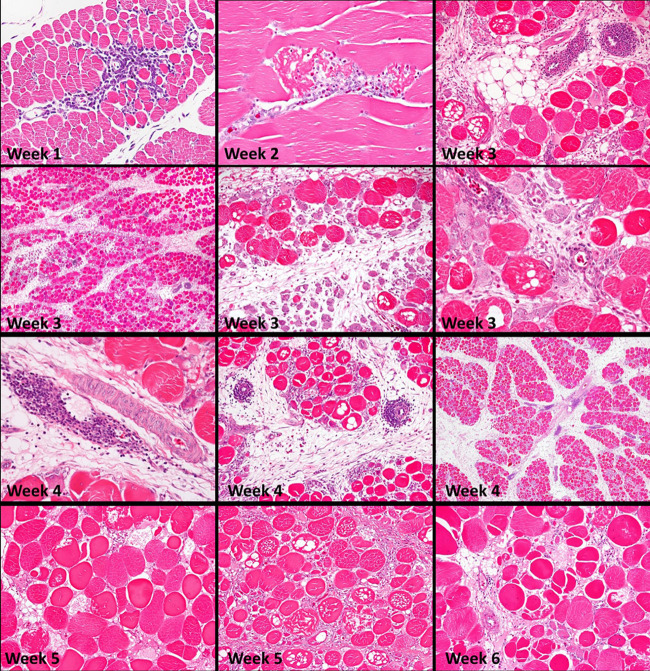
Histopathology, weeks 1 through 6, of superficial pectoral muscles, Study 1. During weeks 1 and 2, the earliest changes are scattered small foci of myofiber degeneration and lymphocytic vasculitis affecting post-capillary venules (phlebitis), which continues to at least six weeks. Muscles are not uniformly affected; lesions are more severe toward the surface of the muscle. Severe muscle degeneration with concurrent regeneration and expansion of the interstitium occurs by three weeks. Lesions intensify during weeks 4 and 5. Vacuolation of affected myofibers is often present, and there is atrophy of muscle fasciculi (bundles). Increasing fibrosis and regeneration begin to predominate through weeks 5 and 6. Connective tissue and fat, contributing to “white striping,” accumulate in interstitial tissues within and between muscle fasciculi. Fiber splitting is prominent at six weeks [Reproduced with permission of Chen et al. (2019)].

White striping (Kuttappan et al., 2013b; Petracci et al., 2013), wooden breast, green muscle, and spaghetti meat have been well described (Chen et al., 2019; Petracci et al., 2019). Histologically, it is difficult to identify differences between cases of wooden breast and striped breast. Wooden breast muscle has lateral packing of collagen and more significant fat infiltration (Velleman et al., 2017). The muscles affected by wooden breast have poor micro vascularization, myodegeneration, rounded fibers, internalization of the nuclei, lymphocytic infiltration, and fibers in the process of regeneration (Soglia et al., 2016; Sihvo et al., 2018; Petracci et al., 2019). These lesions can be observed (Figure 1) since the first week of age (Chen et al., 2019). There is diffuse thickening of the connective tissue of the endomysium and perimysium associated with granulation tissue and increased deposition of connective tissue (fibrosis) and fat deposition or infiltration. Different degrees of muscle damage occur, which are diffuse and not continuous in the *Pectoralis major* muscle (Soglia et al., 2016; Clark and Velleman, 2017).

Several studies have shown that wooden breast is related to hypoxia (Malila et al., 2019) and metabolic disturbances of muscles (Mutryn et al., 2015; Abasht et al., 2016; Petracci et al., 2019). The affected muscles have lower glycogen content, impaired glucose metabolism, higher concentrations of metabolites characteristic of oxidative stress, high protein levels, increased hypoxanthine, xanthine, and urates. The lack of glycogen in the muscle leads to greater activation of the ascorbate biosynthesis pathway and excessive production of hydrogen peroxide. The excess of free radicals can consume glutathione and exacerbate the oxidative process (Mutryn et al., 2015; Abasht et al., 2016). The wooden breast has been observed very early in life (Sihvo et al., 2018; Chen et al., 2019) and frequently broilers affected have increased blood CO_2_ and decreased O_2_ concentrations (Livingston et al., 2019a) related to a state of hypoxia and oxidative stress or low vascularization (Soglia et al., 2016; Sihvo et al., 2018; Chen et al., 2019). Other common findings in broilers affected by wooden breast are increased intracellular calcium (Mutryn et al., 2015) and in white stripping elevated serum levels of creatine kinase, alanine transaminase, aspartate aminotransferase and lactate dehydrogenase (Kuttapan et al., 2013a). Some of these histological, molecular, and metabolic characteristics of myopathies have been observed in poultry hatchlings when incubation conditions have been modified or were suboptimal as it will be discussed in the following sections.

## Evidence of Incubation Effects on Muscle Development and Myopathies

In several studies, it has been observed that variations on temperature during incubation affect myoblast proliferation and skeletal muscle hypertrophy in chickens (Collin et al., 2007; Piestun et al., 2009; Janisch et al., 2015; Naraballobh et al., 2016a, b), turkeys (Maltby et al., 2004; Krischek et al., 2013a), and ducks (Hepp et al., 2006; Hopkins et al., 2011; Liu et al., 2015). Most of the studies have evaluated thermal modifications for the short term during different periods of incubation to stimulate muscle growth. However, suboptimal incubation may affect the occurrence of myopathies. Table 1 presents a summary of some publications that have reported the effects of incubation in myopathies. Myopathies in embryos and newly hatched broilers and ducks have been previously reported (Rigdon, 1967; Rigdon et al., 1968; Yamagiwa et al., 1975).

**TABLE 1:** Overview of literature reporting effects of incubation conditions on myopathy development in chickens, turkeys, and ducks.

Reference	Species	Genetic line	Egg storage (d)	Incubation treatments				Sex	BW		Myopathy	
				T (°C)	RH (%)	Period	Duration		Age (*d*)	Weight (g)	WS	WB
^1^Christensen et al., 2007	Turkeys	Nicholas	4	36.0	53	ED24 – ED28	24 h/day	Mixed	1	x	=	
			4	39.0	53	ED24 – ED28	24 h/day	Mixed	1	x	↑^3^	
^1^Oviedo-Rondón et al., 2016, partially published by Chen et al., 2019	Broilers	Cobb MX x Cobb 500 Ross 344 x Ross 708 Hubbard M99 x Cobb 500	3	37.8	50	ED0 – ED21	24 h/day	Males Females	56	4,345 4,554 4,464	=	=
		Ross 344 x Ross 708 Hubbard M99 x Cobb 500	3	37.0	50	ED0 – ED3	24 h/day	Males Females	56	4,478 4,437	=	=
				39.0	50	ED18 – ED21	24 h/day					
		Cobb MX x Cobb 500	3	37.0	50	ED0 – ED3	24 h/day	Males Females	56	4,410	=	↑
				39.0	50	ED18 – ED21	24 h/day					
^1^Clark et al., 2017	Broilers	Ross 708	x	37.8	53	ED0 – ED21	24 h/day	x	63	5,478	=	
				39.5	65	ED14 – ED18	3 h/day	x	63	5,490	=	
				39.5	65	ED14 – ED18	12 h/day	x	63	5,320	↓^3^	
^1^Livingston et al., 2019b	Broilers	Ross 708	1 – 7	37.8	x	ED0 – ED21	24 h/day	Males	42	3,056	=	=
			8 – 14	37.8	x	ED0 – ED21	24 h/day	Males	42	3,056	↑	=
^2^Nyuiadzi et al., 2020	Broilers	Ross 308	3	37.6	56	ED0 – ED21	24 h/day	Males	40	2,769	=	=
				15.0	81	ED18 – ED19	30 min/day	Males	40	2,818	↑	=
Velleman, Wineland, and Oviedo (*Data not published*)^2^ Da Costa et al., 2016	Ducks	Maple Leaf	4	37.8	50	ED12 – ED21	24 h/day	Males Females	35	3,211	=	=
				37.8^4^	50	ED12 – ED21	24 h/day	Males Females	35	3,139	↑^3^	
				37.8−35.5	50	ED12 – ED21	24 h/day	Males Females	35	3,237	↑^3^	
				37.8−35.5^4^	50	ED12-ED21	24 h/day	Males Females	35	3,240	↑^3^	

*T; Temperature, RH; Relative Humidity, ED; Embryonic Day, x; not reported, =; No significant differences, ↑ and ↓; Increase or decrease incidence or score of myopathies. ^1^Treatments based on eggshell temperature. ^2^Treatments based on machine temperature. ^3^Histological scores of myopathies. ^4^Reduced eggshell conductance at ED14 by immersing one-third of the egg in warm paraffin to reduce the respiratory surface.*

Recently, Nyuiadzi et al. (2020) reported slightly less white striping occurrence at D40, when Ross 308 chicken embryos from 36-wk-old breeders, incubated at constant 37.6°C (99.68°F) and 70% RH were exposed to 15°C and 81% RH for 30 minutes at days 18 and 19. This treatment also had improvements in final body weight (37 grams) compared to the control, but no effects on feed efficiency, carcass and cut up part yields, or meat quality. However, when those chickens were exposed to initial low brooding temperatures (29°C), compared to control brooding (32°C at D1), the white stripping in males tripled, and the footpad dermatitis quadrupled. The environmental conditions post-hatch may affect the responses of hatchlings to incubation treatments. The treatment reported by Nyuiadzi et al. (2020) was a short cold exposure that could occur when transferring eggs from setters to hatchers. As far as brooding conditions are adequate, it seems reducing embryo metabolic heat during this period (ED18 and ED19) is positive to reduce white striping. Still, adaptation to cooler brooding could be an issue. It remains to be evaluated the effects of appropriate stepdown temperatures in the hatchers, as eggshell temperature raises due to embryo metabolic heat production. The reduction in machine temperature is a common practice in single-stage incubation (Boleli et al., 2016), but not used in multi-stage machines or the experiment reported by Nyuiadzi et al. (2020).

The most common situation under industrial conditions is the overheating and reduced egg air exchange that embryos can encounter during the last phase of incubation. This condition can arise either in multi-stage machines or single-stage machines with management and maintenance issues (French, 1997; Boleli et al., 2016). Under commercial conditions, in large incubators, variations in fan speed and direction and or changes in turning angles of egg trays reduce airflow over the eggs (French, 1997) and airflow direction (Van Brecht et al., 2005). The most frequent overheating occurs when all embryos in a machine have the fastest growth rate, metabolic heat production, higher oxygen demand, and CO_2_ production during the plateau of oxygen consumption (Rahn, 1981; Wineland et al., 2006a, b; Christensen et al., 2007). Hypoxia in the last 72 to 96 h of incubation is also more frequent in embryos from eggs stored for more than 5 days due to a delay in development and the extended period between internal and external pipping (Tona et al., 2003a). Livingston et al. (2019b) reported an increased white stripping score (2.58 *vs*. 3.15) when 42-day-old Ross-708 broilers coming from eggs stored for 8 to 14 days were compared to broilers from eggs stored for 1 to 7 days fed *ad libitum*.

### High Temperatures and Low Oxygen Concentration During Incubation in Poultry Myopathies

The high temperatures with >38°C of eggshell temperature, and low oxygen concentrations (<18%) in the incubators during these last stages of incubation can affect the development and viability of muscle fibers in avian species (Table 1). Mulder et al. (1998) and Crossley et al. (2003) demonstrated that when chicken embryos experience hypoxia during the last 3 days of incubation, there is a rearrangement of blood flow, increasing to heart and brain and reducing to liver, muscle and yolk sac. During acute hypoxia, in the plateau of oxygen consumption (Rahn, 1981), the systemic secretion of catecholamines results in vasodilation of the chorioallantoic membrane (CAM) vessels via β-adrenoceptors. In the meantime, vasoconstriction is produced in some of the intraembryonic vessels via α-adrenergic receptors (Crossley et al., 2003). As CAM blood flow accounts for 20 to 50% of total cardiac output (Mulder et al., 1998), CAM vasodilation during hypoxia could cause general embryo hypotension and lower tissue oxygenation.

**FIGURE 2:**
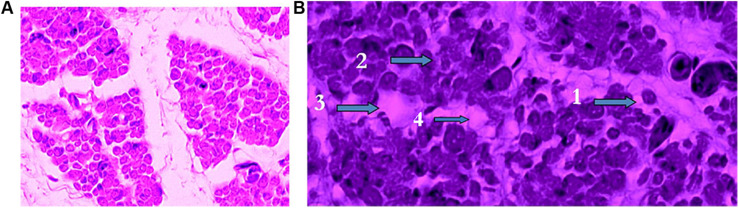
Effect of temperature and oxygen concentration during the last 4 days of incubation on breast muscles of Ross 308 chicks at hatch. **(A)** Breast muscle of chicks incubated at 36°C and 23% of oxygen exhibiting healthy muscle fibers. **(B)** Breast muscle of chicks incubated at 39°C and 17% of oxygen with four stages of muscle degeneration: 1. Rounded fibers, 2. Opaque fibers, 3. Degeneration, and 4. Phagocytosis. (Reproduced with permission of V. Christensen and M. Wineland).

The damages of hypoxia in the breast muscle under high temperatures of incubation have been detected on the first day of age in turkeys (Christensen et al., 2007) and broilers (*unpublished data*). High machine temperatures (39°C *vs*. 36°C) and low oxygen concentrations (17% *vs.* 21%) affected negatively pipping and thigh muscle weights, but only the temperature significantly influenced breast muscle weights and fiber diameters. High temperatures (39°C) increased creatine kinase and lactate dehydrogenase and reduced muscle glycogen. Histologically all signs of muscle degradation (Figure 2) were observed on poults subjected to high temperatures and low oxygen concentrations. These observations are similar to the ones reported during the onset of muscle myopathies (Papah et al., 2017; Chen et al., 2019).

Recently, Halevy (2020) summarized the hypotheses explaining the effects of thermal stress during the early growth phase. Thermal stress during the perinatal period could cause myodegeneration. In these muscles, increased interstitial fibro-adipogenic progenitors and recruitment of myofibroblasts and adipogenic cells are observed. Finally, there is a reduction in the number of satellite cells leading to ineffective myofiber function (Patael et al., 2019). More fibrous and adipose tissue causes a decrease in the endomysial and perimysial connective tissue space that may restrict muscle vascularization (Dransfield and Sosnicki, 1999). Less capillarity limits nutrient and oxygen supply and hampers the removal of metabolic end products like lactic acid.

The deleterious effects of suboptimal incubation on early muscle development have an impact on the incidence and severity of wooden breast at the time of the processing of broilers (Oviedo-Rondón et al., 2016). Specific details of the experiment that will be discussed in the following lines have been partially published by Chen et al. (2019). Breast myopathies were observed in one experiment conducted to evaluate the effects of the genetic line, temperature incubation profiles, and gender. A total of 1,000 eggs from three genetic line crosses: Cobb MX x Cobb 500, Ross 344 x Ross 708, and Hubbard M99 x Cobb 500 were randomly distributed into four machines with two incubation temperature profiles. These eggs were collected from three breeder flocks of 48 weeks of age and stored for 3 days before incubation. In two incubators, the standard eggshell temperature was maintained close to 37.8°C (100.0°F) during the whole incubation period to simulate single-stage incubation (Standard). In the other two machines, eggshell temperatures were low (36.9°C) for the first 3 days and close to 37.8°C until the last 3 days when eggs were subjected to elevated (38.9°C) eggshell temperatures (Low-high), as it is often observed in multi-stage machines. The relative humidity was 50% for multi-stage machines and varied from 65% to 40% in single-stage incubation. Eggshell temperatures were measured five times per day with pipe-probes and thermistors to avoid opening the machines or affecting ventilation. At hatch, 960 chicks sexed were randomly distributed into 80 pens (12 chickens/pen) with five replicate pens per treatment combination. The chicken density was nine chickens/m^2^. All chickens were fed typical corn-soybean meal diets *ad libitum* formulated according to Aviagen recommendations (2014). The lighting program was 23L:1D during the first week and 16L:8D until the end of the experiment. No significant differences (*P* > 0.05) among genetic lines were observed on body weight. At 56 days of age, the average body weight was 4.45 ± 0.08 kg for mixed-sex broilers. At 57 days of age, two broilers per pen were processed, wooden breast and white striping myopathies evaluated 16 h after deboning.

**FIGURE 3:**
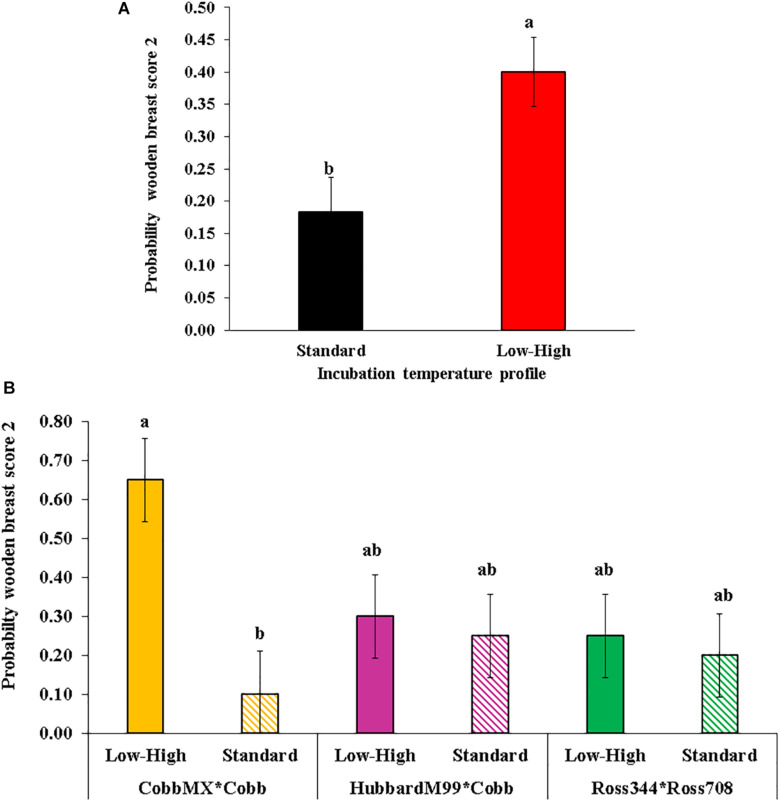
Effect of incubation temperature profile on the probability of observing wooden breast score 2 (moderate severity). **(A)** Effect of incubation temperature profile independently of the genetic line. **(B)** Effect of incubation temperature profile by genetic line cross. [Modified from data of Chen et al. (2019)].

The results indicated that no (*P* > 0.05) three-way interactions were detected. Genetic by temperature profile and genetic by gender interaction effects on white striping (*P* < 0.05) and differences due to genetics (*P* < 0.001) on wooden breast were observed. The most severe scores for wooden breast and white striping were found in Ross 344 x Ross 708 broilers. However, this Ross line had almost 2% points more of breast meat yield (38.50%) than the other two lines (36.78 and 35.03%) at 57 days of age. The lowest scores for white striping were observed on Hubbard M99 x Cobb 500 when broilers were incubated on the standard temperature profile. The females of this line also had the lowest white striping scores as compared to Ross females or Cobb MX x Cobb 500 males. The low-high incubation treatment had 2.18 times more broilers exhibiting wooden breast score 2 than broilers coming from the standard incubation, independently of the genetic line and sex (Figure 3). In Cobb MX x Cobb 500 broilers, the incidence increased six-fold due to low-high suboptimal incubation (0.10 *vs.* 0.65). Still, no significant effects of incubation were observed in the other two genetic lines. The differences in results between genetic lines could be due to thicker and less porous eggshells with low *G* in the Cobb line. It has been demonstrated that eggs from these genetic lines vary on *G* (Oviedo-Rondón et al., 2008a, b). In conclusion, genetic lines differed on white striping, and wooden breast severity and incubation temperature profiles influenced the incidence of myopathies. Deep pectoral myopathy was only observed in a few broilers with no relation to genetic line, incubation profile, or sex. No spaghetti muscle was detected in this experiment.

In contrast, Clark et al. (2017) evaluated the effects of increasing the setter temperature from 37.8°C (100.04°F) to 39.5°C (103.1°F) for 3 and 12 h per day from 14 to 18 days of incubation on time of hatch, growth, breast muscle development and myopathies at 63 days of age. During the last 3 days of incubation, the temperature was stepped down to 36.4, 36.0, and 35.8°C on ED19, 20, and 21, respectively. Chickens were raised in floor pens for a final density of 41.5 kg/m^2^. During the first week, chicks were subject to 23 h of light and then18 h of light for the remainder of the study. Ambient temperatures were controlled as follows: 31 to 29°C for wk 1, 29 to 27°C for wk 2, 27 to 24°C for wk 3, 24 to 22°C for wk 4, 22 to 21°C for wk 5, 21 to 20°C for wk 6, 20 to 18°C for wk 7, and 18°C for wk 8 and 9. The chicks incubated at an increased temperature for 12 h per day had reduced (*P* < 0.01) body and carcass weights (∼150 g less) compared to the 3 h and control treatments, and worse (*P* < 0.01) feed conversion ratio (1% worse). But, this treatment applied 12 h per day had fewer broilers with moderate and severe myopathies compared to the control. Myopathies were evaluated by a microscopic myopathy score system based upon the severity of necrotic and fibrotic attributes, as described by Clark and Velleman (2017). The authors concluded that increasing incubation temperature from ED14 to ED18 was a feasible management strategy to reduce myopathies while maintaining meat quality parameters. The authors also evaluated the time of hatch. Chickens that hatched early had a higher occurrence of moderate to severe degenerative changes in the breast muscle compared to those that hatched later. The longer time that these chickens had to reach feed probably affected satellite cells. Even though the eggshell temperature was not measured in that experiment, it is known that machine temperatures higher than 37.6°C after 15 days of incubation could cause overheating in the embryos (Molenaar et al., 2013), and this slight overheating could explain the effects on live performance observed in this experiment.

The differences in the responses observed between the two experiments discussed here are related to the length of exposure to higher temperatures (3 days *vs*. 12 h), and the timing of application of the heat stress (14 to 18 days *vs*. 18 to 21 days). Chicken embryos respond differently to heat stress (Molenaar et al., 2013) and hypoxia (Crossley et al., 2003) depending on timing during embryo development, being more critical and deleterious for muscles during the last phase of incubation (Hartley et al., 1992; Christensen et al., 2007; Boleli et al., 2016; Halevy, 2020).

### High Temperatures and Reduced Eggshell Conductance Effects in Ducks

**FIGURE 4:**
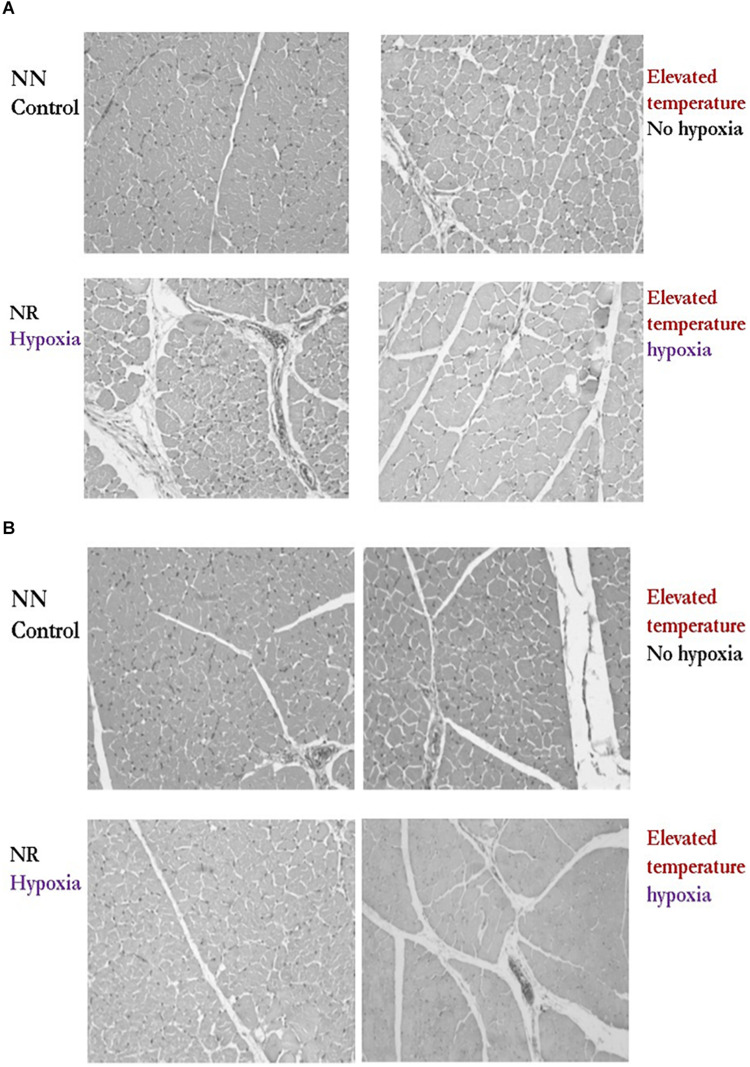
Effect of temperature after 12 days of incubation (Normal *vs*. Elevated) and reduced eggshell conductance (Normal *vs* Reduced) after 14 days of incubation on breast muscle histomorphology of drake **(A)** and hen **(B)** Pekin ducks at 35 days of age. These results demonstrated altered muscle fiber diameter with increased perimysial and endomysial connective spacing compared to the control. (Reproduced with permission of M. Wineland. S. Velleman and E. Oviedo).

Some incubation effects on muscle myopathies have also been observed in Pekin ducks. One experiment was conducted to evaluate the effects of *G* and temperature profiles during incubation on duck muscle histological characteristics at 35 days of age. The materials and methods and a portion of the results of this experiment were published by Da Costa et al. (2016). Briefly, a total of 10,000 Pekin duck eggs were randomly sorted, equally distributed into four groups, and placed in two single-stage incubators under commercial conditions. Treatments consisted of two *G*, reduced and normal, and two incubation temperature profiles, elevated and normal, after ED12. Eggshell *G* was reduced by dipping lower one-third of eggs in wax at 14 days of incubation. At hatch, ducklings were placed in a commercial house. At hatch and market age, five drakes and five hens of each treatment combination were selected, weighed, and processed. The histomorphological structure of the muscle was assessed for muscle fiber integrity, perimysial, and endomysial spacing, as described in Velleman et al. (2003). For the histological data, a 2x2 factorial design with temperature and *G* as main factors was used. Results (Figure 4) indicated that either elevated T or reduced *G* altered (*P* < 0.05) muscle fiber diameter and increased perimysial and endomysial fibrosis. The reduced *G* treatment had the most detrimental effects with increased collagen deposition typical of fibrosis in the perimysial connective tissue space and the presence of inflammatory cells suggestive of myofiber necrosis. Interestingly, elevated temperature with both hypoxia and without hypoxia caused by variations on eggshell *G* increased the endomysial spacing between the myofibers compared to the control. Unlike the males, the hens with hypoxia (reduced *G*) did not have altered collagen deposition typical of fibrosis. In the hens, the elevated temperature with hypoxia decreased muscle fiber bundle size and the endomysial spacing between individual muscle fibers. Decreases of this nature are associated with reduced muscle mass accretion.

### Metabolic Changes in Poultry Embryo Muscle Post Environmental Stress

Suboptimal environmental conditions during incubation can cause metabolic modifications in the muscle that may continue in the life post-hatch. To understand the relationship between incubation effects and myopathies is indispensable to review the metabolic changes observed in myopathies. The histological (Velleman et al., 2017; Sihvo et al., 2018), metabolic characteristics (Abasht et al., 2016), the gene expression (Mutryn et al., 2015), and the chronological evolution (Chen et al., 2019) of the lesions in the muscles with wooden breast in chickens are very similar to those observed in the muscles of human and diabetic animals (D’Souza et al., 2013). Chronic low-grade inflammation, oxidative stress, lower vascularization, and impaired extracellular matrix remodeling are common denominators for mechanisms underlying deterioration of chicken muscle health and decreased satellite cell functionality similar to the observed in diabetes mellitus type 2 (Lake and Abasht, 2020). These failures in avian muscle development are likely due to disturbances in glucose metabolism, or insulin resistance. The insulin response in avian species differs from metabolism in mammals, with different transport proteins that respond to insulin (Seki et al., 2003) and a cellular system that causes the birds’ muscles to be insensitive to insulin despite the high plasma glucose levels (Dupont et al., 2009). This control system could be altered by high levels of corticosterone induced by stressors (Dong et al., 2007; Yuan et al., 2008; Zhao et al., 2009). These high corticosterone levels can be observed during the perinatal period in avian species, and chicken embryos could be induced to have diabetes type I (Shi et al., 2014). The metabolic changes in the muscle caused by other poultry myopathies have not been described with the same detail given to wooden breast.

High temperatures and oxygen concentrations during the last phase of incubation affect the metabolism of the chicks, especially in the glucose and insulin balance (Loyau et al., 2015). Increased hatcher temperature elevates embryonic plasma glucose concentrations compared to controls (Molenaar et al., 2013), which was accompanied by increased plasma glucagon and variations in IGF-I concentrations (Christensen et al., 2001). IGF-I also can be stimulated by oxygen supplementation during incubation and decreased by hypoxia (Christensen et al., 1999). These changes appeared dependent on the genetics of embryos. Embryos selected for fast growth show more fluctuation in response to environmental oxygen and temperature than embryos from genetics selected for egg production. In the same way, the embryos from eggs with low *G* are more susceptible to changes in environmental conditions (Oviedo-Rondón et al., 2008a, b).

Al-Musawi et al. (2012) demonstrated with molecular and histological methods that increasing incubation temperature from 37.5 to 38.5°C between ED 4 and ED7 enhanced motility and body mass in both layer and broiler embryos. However, the muscle responses vary between layers and broilers. In layers, increased mRNAs were observed in hindlimb Myf5 at ED5–8, Pax7 (ED5–10), Bone Morphogenetic Protein 4, BMP4 (ED6–9) and IGF-I (ED9–10, ED18). These molecular changes led to gastrocnemius muscle hypertrophy, increased fiber and nuclei numbers, and higher nuclei to fiber ratio by ED18. In contrast, broiler embryo hindlimbs had a delay in the peak of mRNA Myf5 expression, increased Pax7 (ED5, ED7–10), and BMP4 (ED6–8), increased proliferator-activated receptor gamma (PPARγ) mRNA (ED7–10) but reduced IGF-I (ED8–10). The result in broilers was a lower gastrocnemius cross-sectional area with fewer fiber and nuclei, no changes in fiber to nuclei ratio (ED18), and increased intramuscular lipid deposition. These authors concluded that incubation temperature modifications affected broiler and layer embryos differently. The molecular regulation of hindlimb myogenic, adipogenic, and growth factor expression varied between these two groups causing different muscle phenotypes. Lipid infiltration and metabolic muscle changes are common in myopathies (Papah et al., 2017). Increasing incubation temperature in one degree Celsius, from ED4 to ED7, created all these metabolic changes in muscles. Heat stress during the last stage of incubation cause even more significant impacts, because higher metabolic heat release and oxygen demand are critical for muscle development.

Chicken IGF-I has been recognized as a candidate gene responsible for body composition, growth, fat deposition, and other metabolic activities critical for satellite cell proliferation and skeletal muscle hypertrophy (Zanou and Gailly, 2013). The production of IGF-I is highly dependent on oxygen concentrations (Duan et al., 2010). The beneficial effects of IGF-I lowering glucose via inhibition of renal gluconeogenesis (Clemmons, 2007) and sensitizing cells for insulin (Kabir et al., 2010) can aid in glucose metabolism regulation. Low IGF-I, hyperglycemia, and failures of glucose metabolism are frequently observed in myopathies like striated and wooden breast muscle (Abasht et al., 2016; Lake and Abasht, 2020).

Hyperglycemia has a negative effect on vascularization. Larger et al. (2004) demonstrated that hyperglycemia decreases angiogenesis in the chicken CAM model after the 5th day. Two days after inducing hyperglycemia, these authors detected increased apoptosis of endothelial cells and pericytes, using transferase-mediated deoxyuridine triphosphate nick-end labeling assay and electron microscopy. The analysis of bromodeoxyuridine incorporation revealed a reduction in endothelial cell proliferation without altering the gene expression of vascular growth factors. In summary, hyperglycemia can impair angiogenesis through induction of apoptosis and decreased proliferation of endothelial cells. Lack of vascularization in the breast muscle is one of the initial signs of myopathies (Sihvo et al., 2018; Chen et al., 2019; Lake and Abasht, 2020). It remains to be proven that vascularization can be reduced in breast muscle after stressful conditions in the last phase of incubation.

Finally, satellite cells are multipotential stem cells that mediate post-hatch muscle growth and respond differently to temperature based upon aerobic versus anaerobic fiber-type origin. Harding et al. (2015) observed that *Pectoralis major* and *Biceps femoris* satellite cells accumulated less lipid when incubated at low temperatures and more fat at elevated temperatures compared to the control. These researchers did not detect significant differences in apoptosis. Breast muscles, which are rich in type IIB fast-twitch glycolytic anaerobic fibers, can be more susceptible to high environmental temperatures and predisposed to adipogenic conversion than other muscles. Satellite cells isolated from the *Pectoralis major* accumulated more lipid at elevated temperatures compared to *Biceps femoris* cells. The higher lipid content could be related to the higher expression of adipogenic genes such as CCAAT/enhancer−binding protein β (C/EBPβ) and PPARγ in satellite cells isolated from the *Pectoralis major* compared to those from *Biceps femoris* (Harding et al., 2015). This experiment demonstrated that breast muscle during embryo development could be more susceptible to develop the fat infiltration observed in breast myopathies (Papah et al., 2017; Sihvo et al., 2018; Chen et al., 2019; Petracci et al., 2019).

**FIGURE 5:**
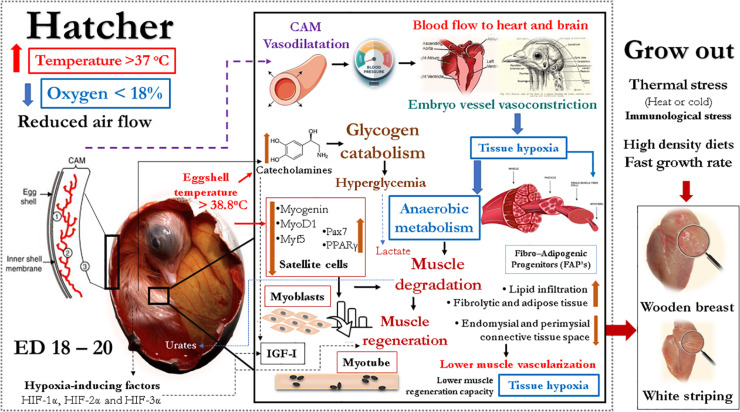
Graphical description of the hypothesis supported by the evidence presented in this manuscript indicating that temperature, oxygen concentrations, and ventilation, especially during the last phase of incubation, affect metabolism of glucose and lipids in the embryonic muscle leading to changes in glucose and fat muscle metabolism, satellite cell number and function, vascularization and capabilities of regeneration. The final result could be susceptibility to develop myopathies during grow out.

In summary, there is evidence in the literature indicating that temperature and oxygen concentrations during incubation could modify muscle glucose metabolism, insulin balance, IGF-I release, muscle adipogenesis, vascularization, and satellite cell numbers and functions that are related to myopathies. These modifications in muscle metabolic responses may make hatchlings more susceptible to develop myopathies during grow out due to thermal stress, high-density diets, and fast growth rates (Petracci et al., 2019). Based on the evidence presented, it is hypothesized that incubation conditions may play an important role in the occurrence of myopathies, and the main steps that could lead to myopathies are described in Figure 5.

## Conclusion

Variations on temperature during incubation impact muscle development post-hatch. Higher temperatures during short periods of 3, 6 and up to 12 h during ED 4 to 7 or 14 to 18 have been shown to have positive effects on muscle growth; however, responses in muscle composition and meat properties vary depending on the genetic line. The last phase of incubation in avian species is critical for muscle development. During this period, stressful conditions due to high temperatures or low oxygen concentrations can alter the maturation processes. Embryos coming from eggs stored for periods more extended than 5 days may be more susceptible to hypoxia and to develop myopathies due to the extended period between internal and external piping. Higher degrees of stress during these stressful conditions at the end of incubation increase corticosterone, catecholamines, reduce IGF-I, and increased glucose levels due to higher degradation of glycogen and increased insensitivity to insulin. This cascade of reactions could affect satellite cell numbers and ability for regeneration post-hatch, reduce vascularization, increase hypoxia, oxidative stress, inflammation, cause impaired extracellular matrix remodeling, switching of type II muscle fibers, and finally cause some apoptosis increasing adipogenesis in the muscles. The combined results of this developmental plasticity to common environmental stress close to hatch could increase the severity and incidence of myopathies observed in breast meat of broilers, turkeys, and ducks at processing age.

## Author Contributions

The information presented was developed during collaborative research with several other groups, including all three authors for several years. EO-R collected all the information and wrote the article. SV and MW reviewed and corrected the manuscript.

## Conflict of Interest

MW is currently a consultant under Hatchery Consultant Inc. The remaining authors declare that the research was conducted in the absence of any commercial or financial relationships that could be construed as a potential conflict of interest.
